# Code Error in “Diagnostic Classification and Prognostic Prediction Using Common Genetic Variants in Autism Spectrum Disorder: Genotype-Based Deep Learning”

**DOI:** 10.2196/66556

**Published:** 2025-05-06

**Authors:** Catriona Miller, Theo Portlock, Denis M Nyaga, Greg D Gamble, Justin M O'Sullivan

**Affiliations:** 1 Liggins Institute, University of Auckland, 85 Park Road, Private Bag 92019, Auckland, 1142, New Zealand, 64 099239868

**Keywords:** autism prediction, machine learning, data leakage

Wang and Avillach [1] developed a convolutional neural network (CNN)–based diagnostic classifier for autism spectrum disorder (ASD). After preprocessing the genomics data from the Simons Simplex Collection (SSC) [2], common variants that may be protective or pathogenic for autism were extracted based on a *χ*^2^ test. The authors then designed a CNN-based diagnostic classifier for ASD with an accuracy and area under the receiver operating characteristic curve of 88% and 0.955, respectively. The predictor in Wang and Avillach [1] is currently considered the exemplar in the field, giving much more accurate predictions for autism than other studies [3].

However, when inspecting the code and repeating the analyses, we contend that the method used is flawed and leads to an approximately 30% overestimation of predictive ability.

Wang and Avillach [1] did not provide a GitHub link to the code that was used in their paper. However, code can be found in Dr Wang’s GitHub repository [4] that matches the results and figures in the manuscript.

An error occurred in the data split for training and test sets. The methods state “...the SSC samples were partitioned into two sets based on random sampling of individuals into a training set (80%) and a hold-out test set (20%). There was no overlap of individuals across the two partitions” [1]. However, the code uses different indexing methods for the test and training sets (Multimedia Appendix 1). Because there appears to be no random seed in Wang and Avillach [1], we cannot reproduce the exact overlap that was mentioned in the manuscript. However, simulations (N=100) using the code identified an average of 80% (SD 1%) of the test dataset being represented in the training dataset.

We corrected this error in the code [5] and generated new models using the 100 features that were identified in Wang and Avillach [1] and the genomics data from the SSC [2]. Simulations (N=100) with these models result in an area under the receiver operating characteristic curve of 0.61 (SD 0.02) and an accuracy of 60% (SD 2%; Multimedia Appendix 1). This is 0.34 and 28% lower than the reported metrics, respectively, in Wang and Avillach [1].

The accuracy of the CNN-based diagnostic classifier for ASD presented in Wang and Avillach [1] is overestimated by ~28%. We contend that Wang and Avillach [1] should be retracted according to the Committee on Publication Ethics (COPE) guidelines.

## Supplementary material

10.2196/66556 Multimedia Appendix 1 Walkthrough of the steps that were taken and the results of our attempt to reproduce the work of Wang and Avillach (2021).

## Abbreviations

ASD: autism spectrum disorder
CNN: convolutional neural network
COPE: Committee on Publication Ethics
SSC: Simons Simplex Collection
