# Supplementary material for: Code Error in “Diagnostic Classification and Prognostic Prediction Using Common Genetic Variants in Autism Spectrum Disorder: Genotype-Based Deep Learning”
Source: JMIR Med Inform. 2025 May 6;13:e66556. doi: 10.2196/66556 (PMC12138136; doi:10.2196/66556)
Supplement: Multimedia Appendix 1 [file medinform-v13-e66556-s001.pptx]

## Slide 1
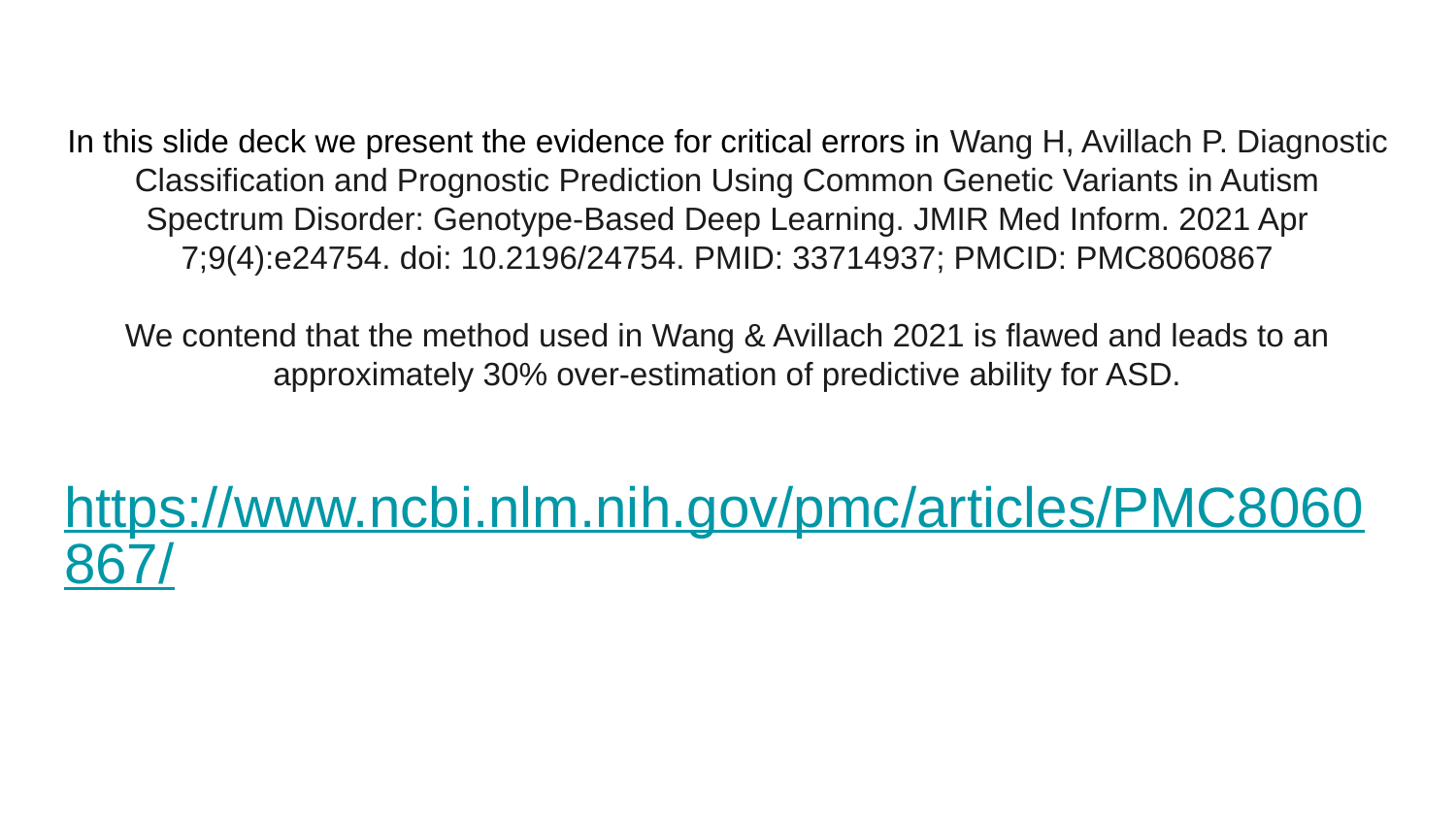

# In this slide deck we present the evidence for critical errors in Wang H, Avillach P. Diagnostic Classification and Prognostic Prediction Using Common Genetic Variants in Autism Spectrum Disorder: Genotype-Based Deep Learning. JMIR Med Inform. 2021 Apr 7;9(4):e24754. doi: 10.2196/24754. PMID: 33714937; PMCID: PMC8060867
We contend that the method used in Wang & Avillach 2021 is flawed and leads to an approximately 30% over-estimation of predictive ability for ASD.
https://www.ncbi.nlm.nih.gov/pmc/articles/PMC8060867/

## Slide 2
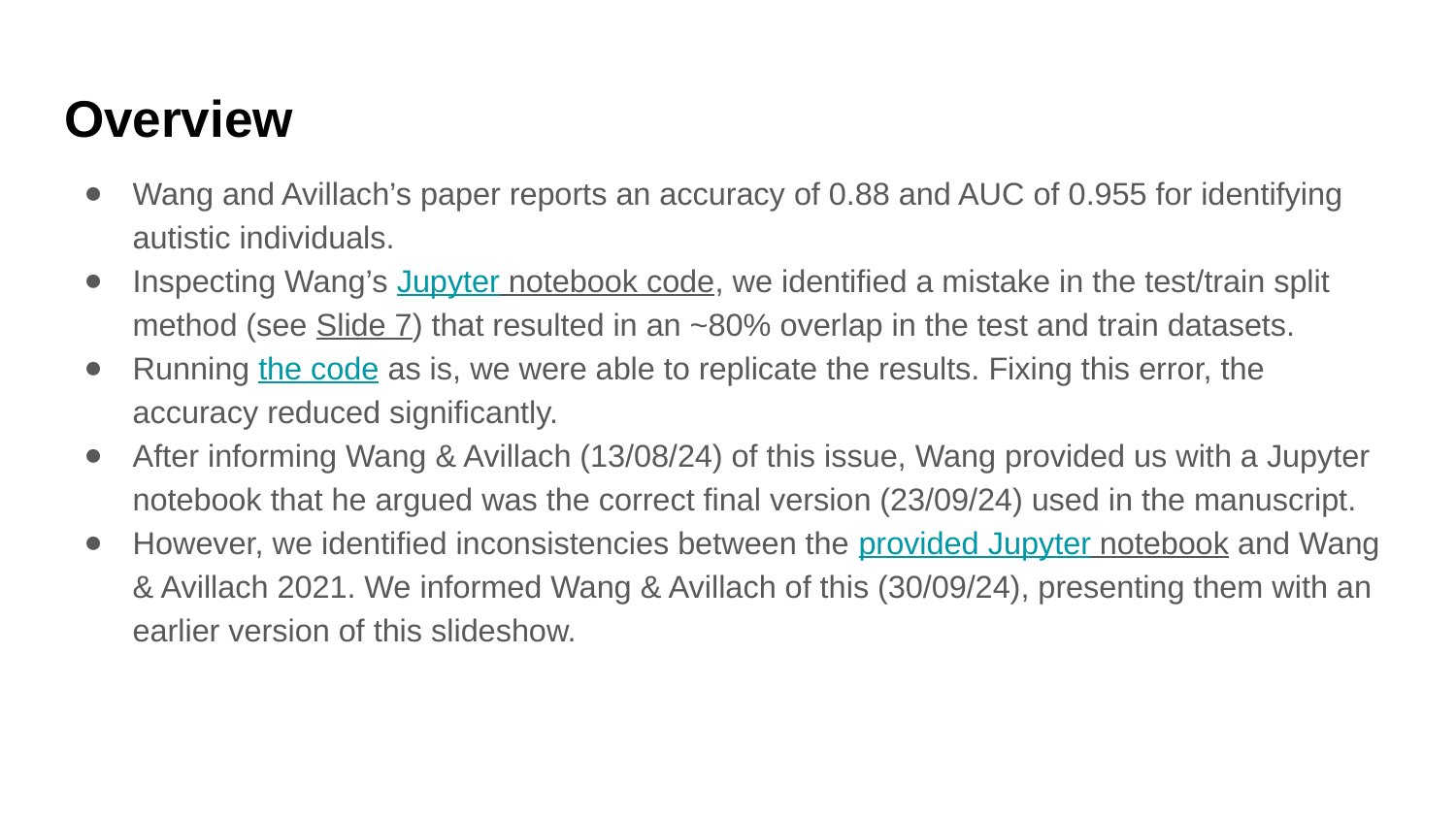

# Overview
Wang and Avillach’s paper reports an accuracy of 0.88 and AUC of 0.955 for identifying autistic individuals.
Inspecting Wang’s Jupyter notebook code, we identified a mistake in the test/train split method (see Slide 7) that resulted in an ~80% overlap in the test and train datasets.
Running the code as is, we were able to replicate the results. Fixing this error, the accuracy reduced significantly.
After informing Wang & Avillach (13/08/24) of this issue, Wang provided us with a Jupyter notebook that he argued was the correct final version (23/09/24) used in the manuscript.
However, we identified inconsistencies between the provided Jupyter notebook and Wang & Avillach 2021. We informed Wang & Avillach of this (30/09/24), presenting them with an earlier version of this slideshow.

## Slide 3
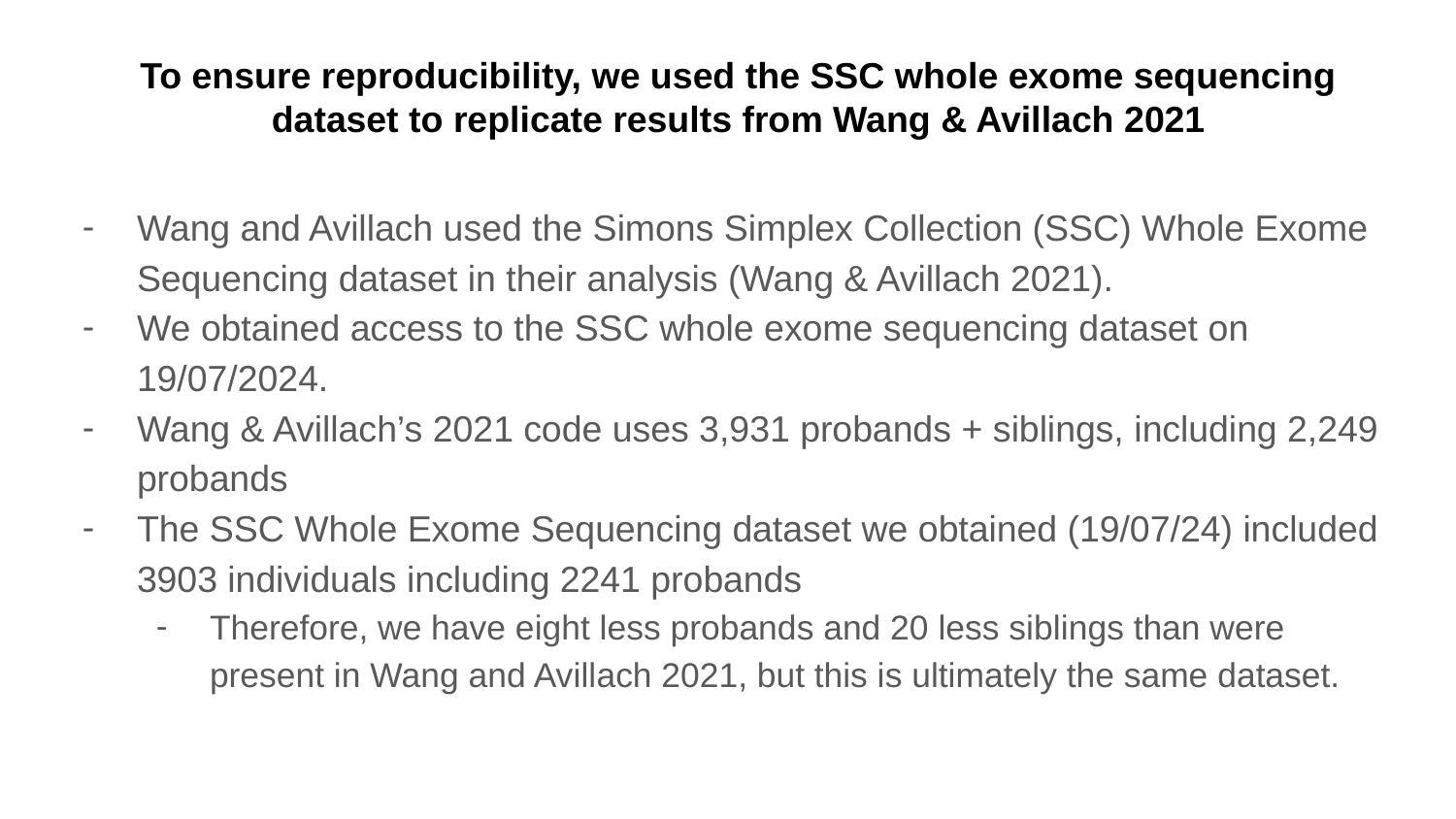

# To ensure reproducibility, we used the SSC whole exome sequencing dataset to replicate results from Wang & Avillach 2021
Wang and Avillach used the Simons Simplex Collection (SSC) Whole Exome Sequencing dataset in their analysis (Wang & Avillach 2021).
We obtained access to the SSC whole exome sequencing dataset on 19/07/2024.
Wang & Avillach’s 2021 code uses 3,931 probands + siblings, including 2,249 probands
The SSC Whole Exome Sequencing dataset we obtained (19/07/24) included 3903 individuals including 2241 probands
Therefore, we have eight less probands and 20 less siblings than were present in Wang and Avillach 2021, but this is ultimately the same dataset.

## Slide 4
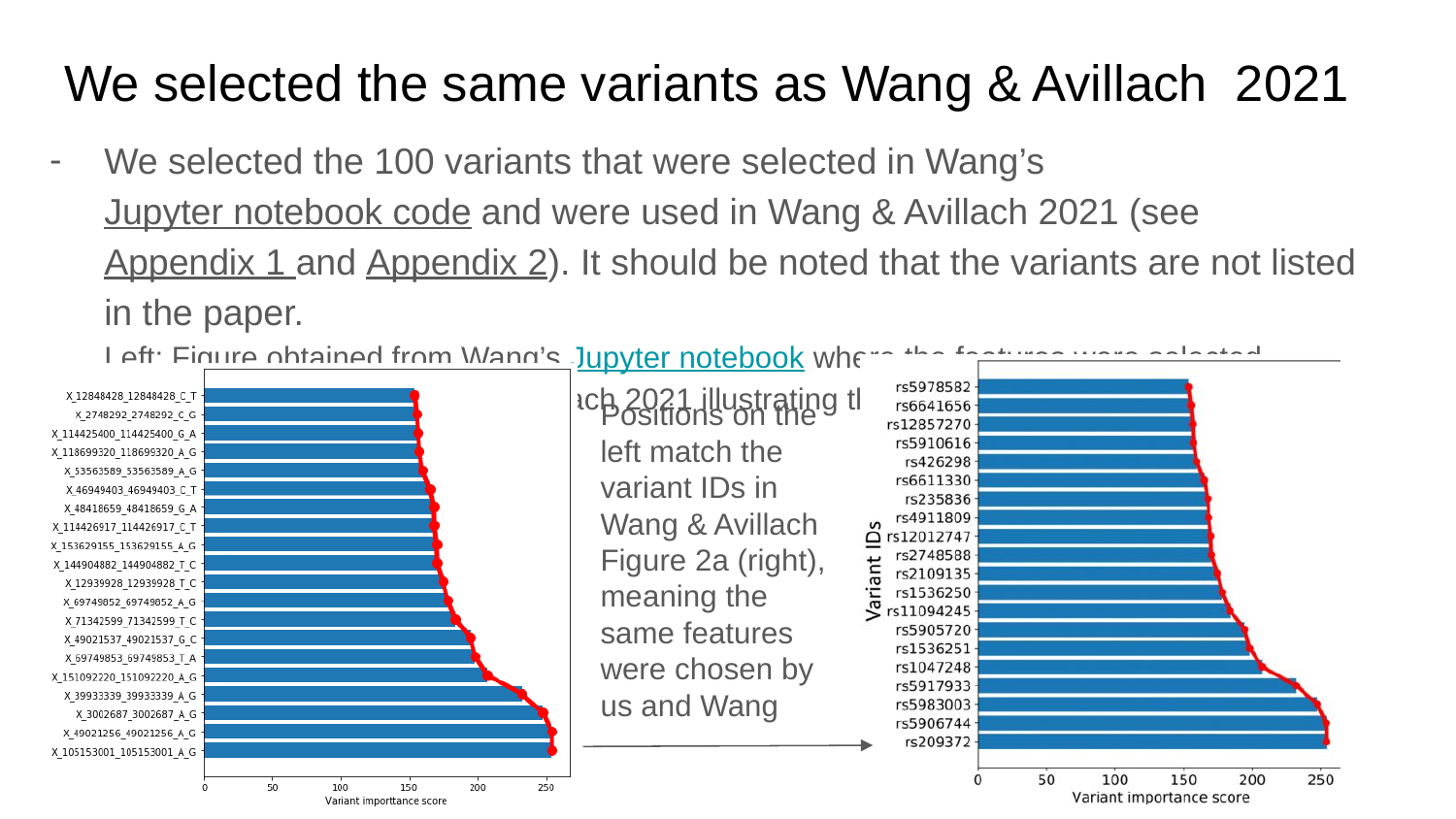

# We selected the same variants as Wang & Avillach 2021
We selected the 100 variants that were selected in Wang’s Jupyter notebook code and were used in Wang & Avillach 2021 (see Appendix 1 and Appendix 2). It should be noted that the variants are not listed in the paper.
Left: Figure obtained from Wang’s Jupyter notebook where the features were selected.
Right: Figure 2a from Wang & Avillach 2021 illustrating the top features they identified
Positions on the left match the variant IDs in Wang & Avillach Figure 2a (right), meaning the same features were chosen by us and Wang

## Slide 5
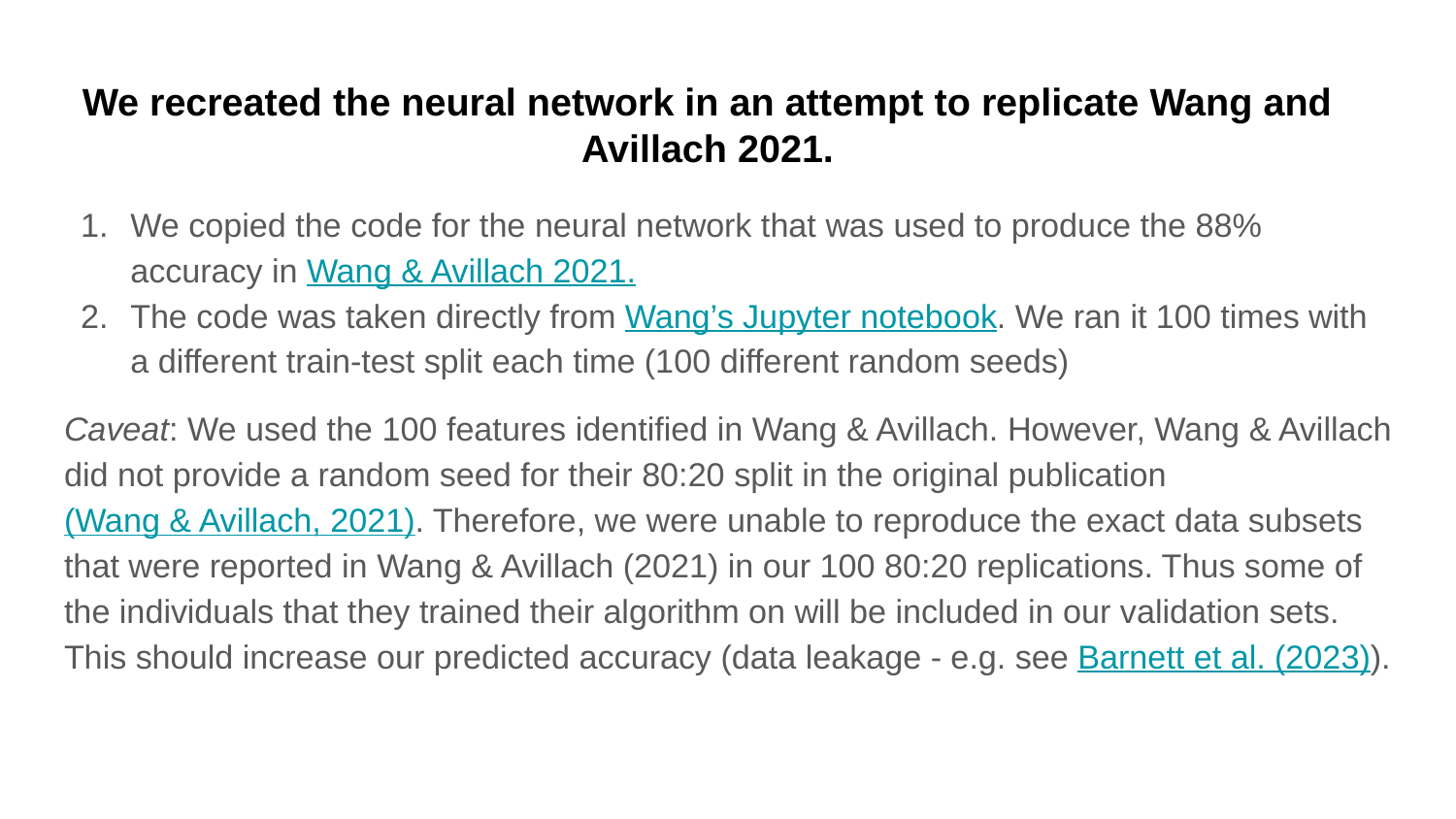

# We recreated the neural network in an attempt to replicate Wang and Avillach 2021.
We copied the code for the neural network that was used to produce the 88% accuracy in Wang & Avillach 2021.
The code was taken directly from Wang’s Jupyter notebook. We ran it 100 times with a different train-test split each time (100 different random seeds)
Caveat: We used the 100 features identified in Wang & Avillach. However, Wang & Avillach did not provide a random seed for their 80:20 split in the original publication (Wang & Avillach, 2021). Therefore, we were unable to reproduce the exact data subsets that were reported in Wang & Avillach (2021) in our 100 80:20 replications. Thus some of the individuals that they trained their algorithm on will be included in our validation sets. This should increase our predicted accuracy (data leakage - e.g. see Barnett et al. (2023)).

## Slide 6
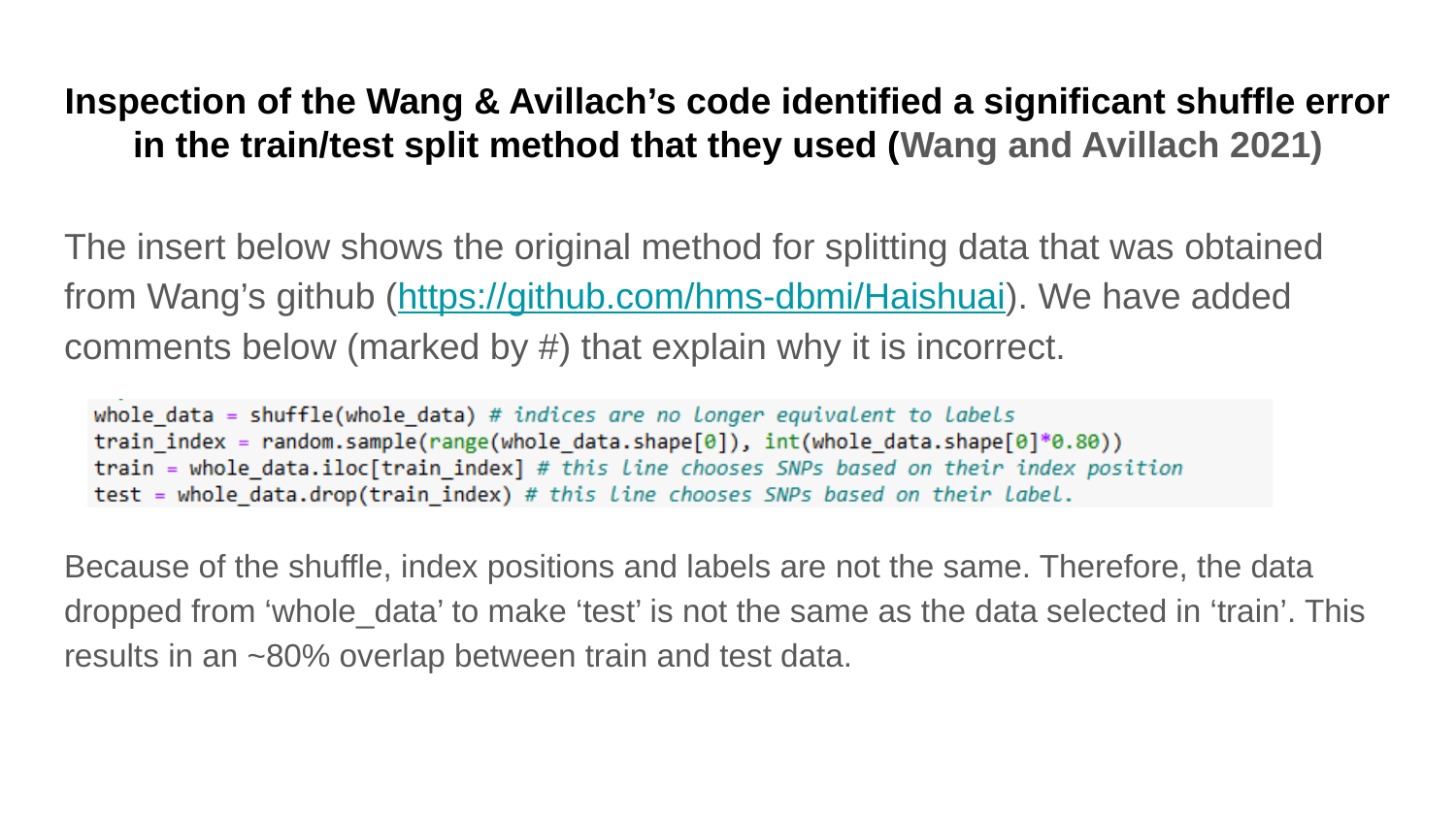

# Inspection of the Wang & Avillach’s code identified a significant shuffle error in the train/test split method that they used (Wang and Avillach 2021)
The insert below shows the original method for splitting data that was obtained from Wang’s github (https://github.com/hms-dbmi/Haishuai). We have added comments below (marked by #) that explain why it is incorrect.
Because of the shuffle, index positions and labels are not the same. Therefore, the data dropped from ‘whole_data’ to make ‘test’ is not the same as the data selected in ‘train’. This results in an ~80% overlap between train and test data.

## Slide 7
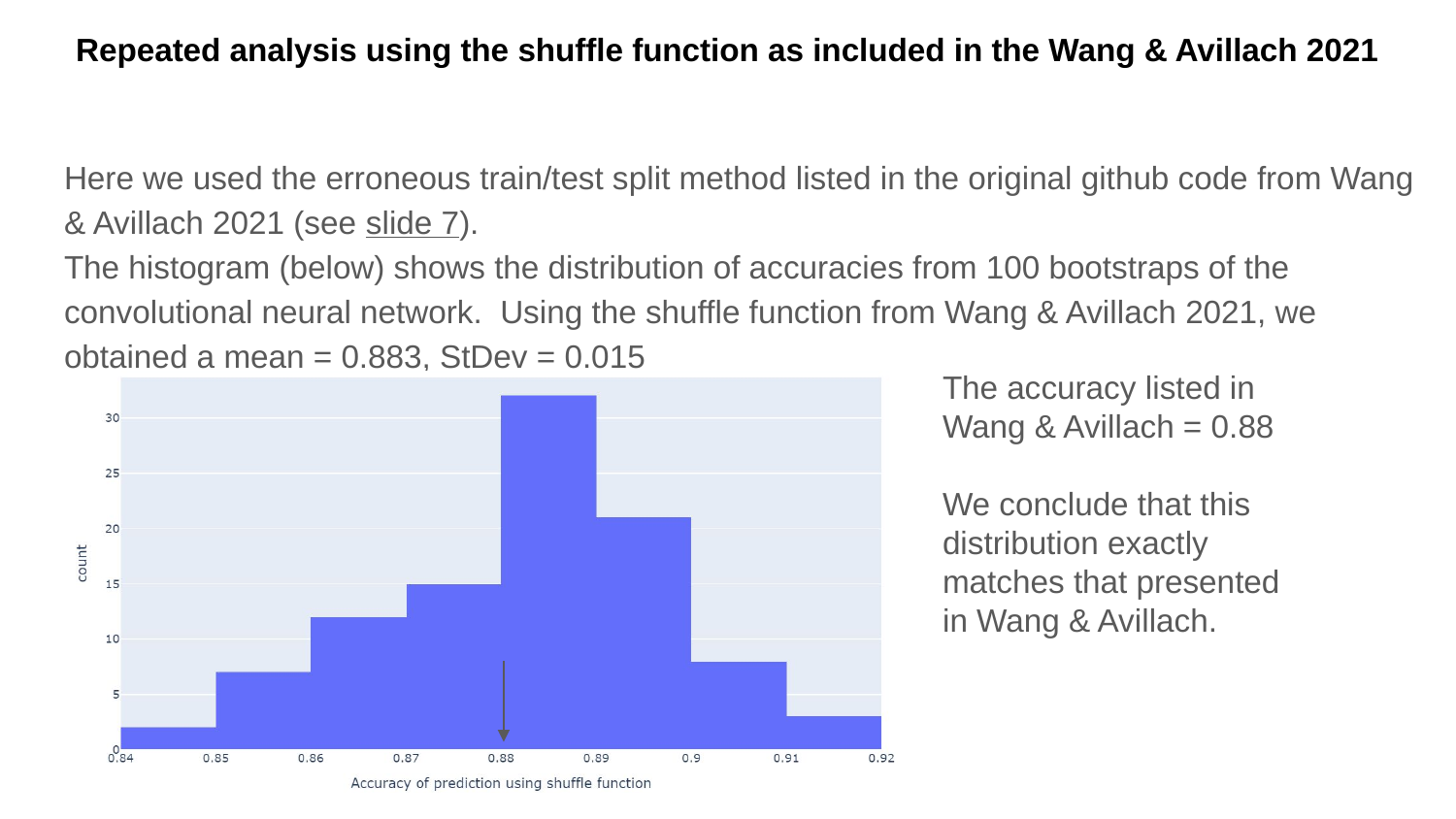

# Repeated analysis using the shuffle function as included in the Wang & Avillach 2021
Here we used the erroneous train/test split method listed in the original github code from Wang & Avillach 2021 (see slide 7).
The histogram (below) shows the distribution of accuracies from 100 bootstraps of the convolutional neural network. Using the shuffle function from Wang & Avillach 2021, we obtained a mean = 0.883, StDev = 0.015
The accuracy listed in Wang & Avillach = 0.88
We conclude that this distribution exactly matches that presented in Wang & Avillach.

## Slide 8
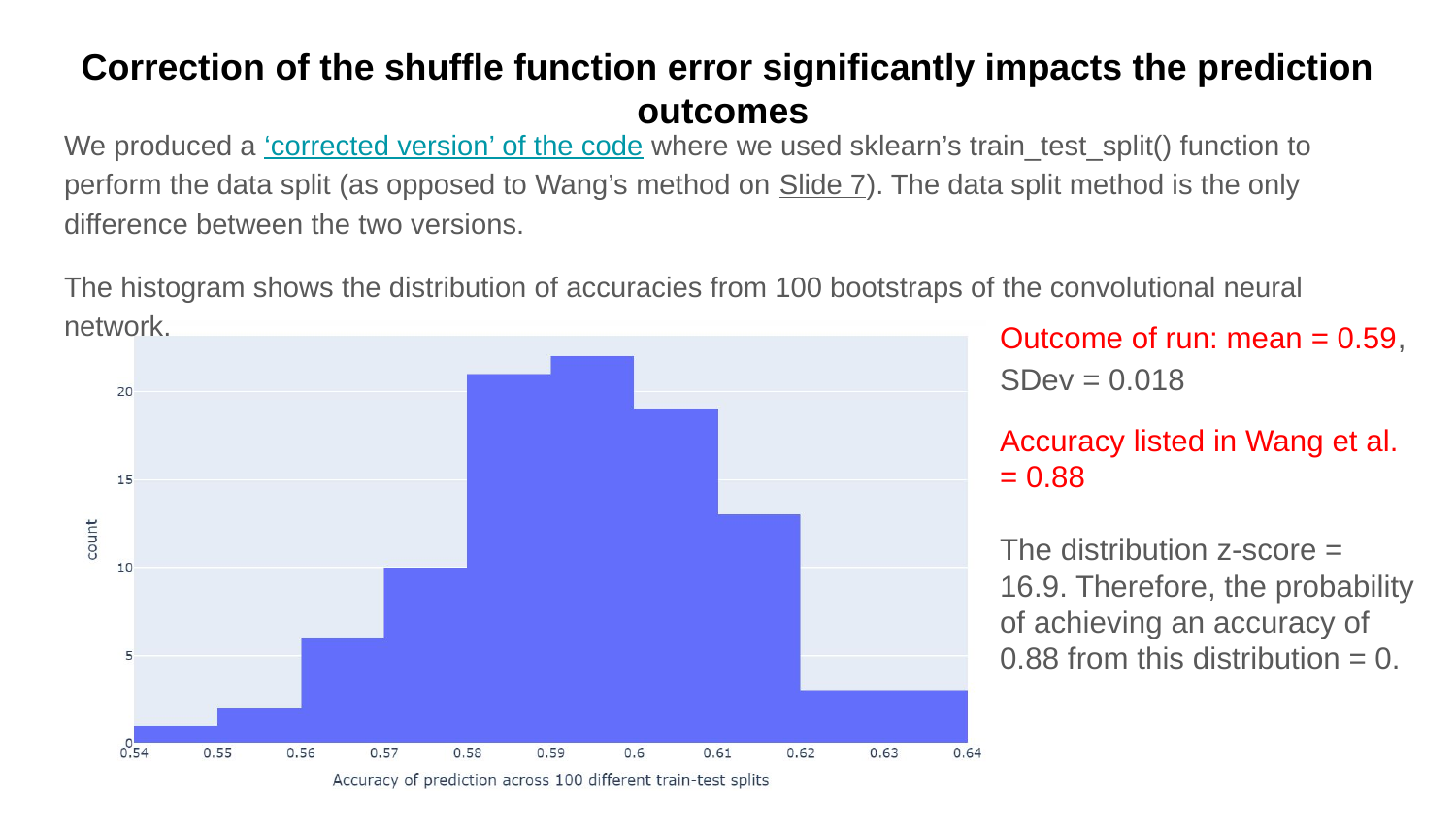

# Correction of the shuffle function error significantly impacts the prediction outcomes
We produced a ‘corrected version’ of the code where we used sklearn’s train_test_split() function to perform the data split (as opposed to Wang’s method on Slide 7). The data split method is the only difference between the two versions.
The histogram shows the distribution of accuracies from 100 bootstraps of the convolutional neural network.
Outcome of run: mean = 0.59, SDev = 0.018
Accuracy listed in Wang et al. = 0.88
The distribution z-score = 16.9. Therefore, the probability of achieving an accuracy of 0.88 from this distribution = 0.

## Slide 9
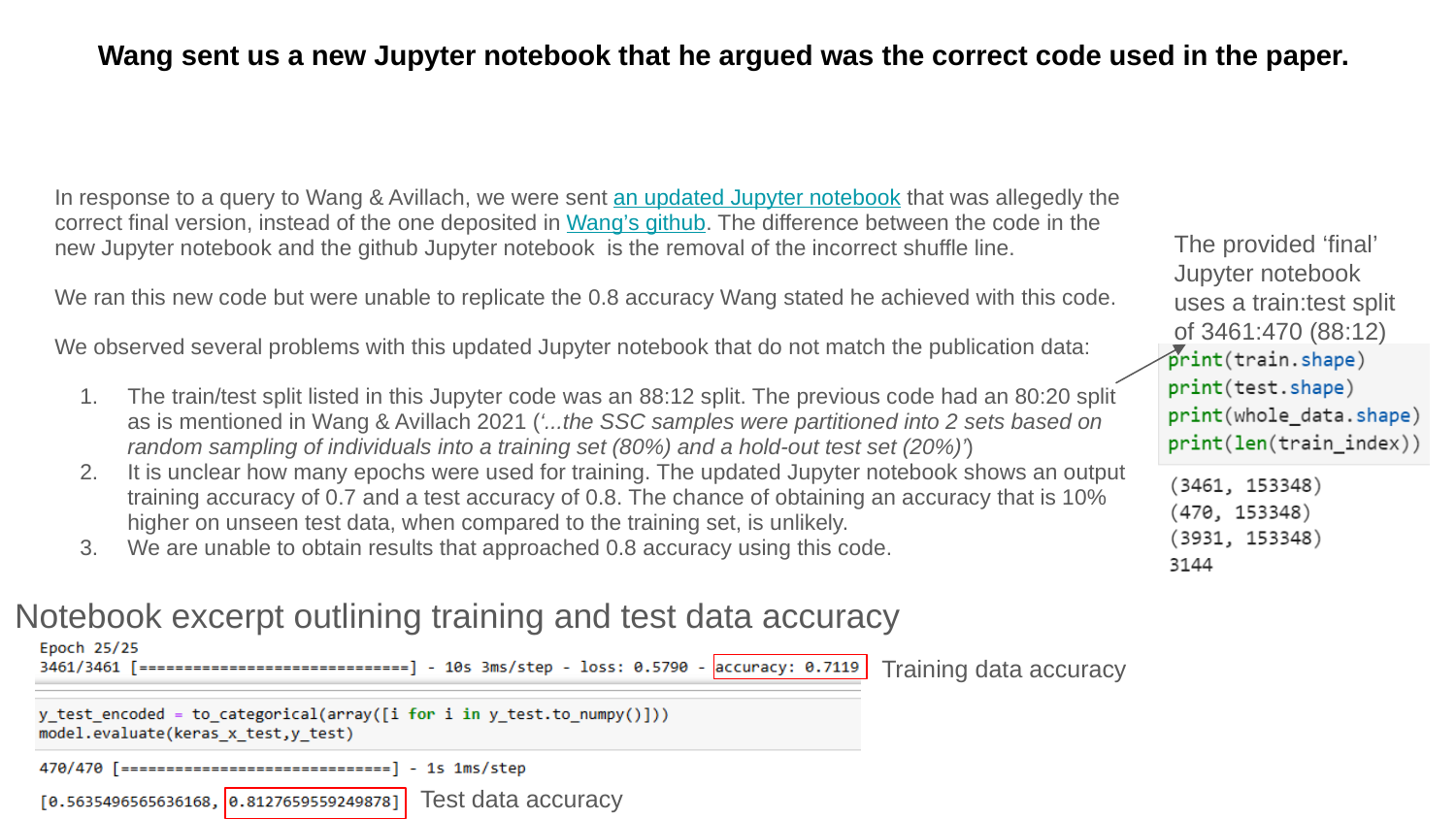

# Wang sent us a new Jupyter notebook that he argued was the correct code used in the paper.
In response to a query to Wang & Avillach, we were sent an updated Jupyter notebook that was allegedly the correct final version, instead of the one deposited in Wang’s github. The difference between the code in the new Jupyter notebook and the github Jupyter notebook is the removal of the incorrect shuffle line.
We ran this new code but were unable to replicate the 0.8 accuracy Wang stated he achieved with this code.
We observed several problems with this updated Jupyter notebook that do not match the publication data:
The train/test split listed in this Jupyter code was an 88:12 split. The previous code had an 80:20 split as is mentioned in Wang & Avillach 2021 (‘...the SSC samples were partitioned into 2 sets based on random sampling of individuals into a training set (80%) and a hold-out test set (20%)’)
It is unclear how many epochs were used for training. The updated Jupyter notebook shows an output training accuracy of 0.7 and a test accuracy of 0.8. The chance of obtaining an accuracy that is 10% higher on unseen test data, when compared to the training set, is unlikely.
We are unable to obtain results that approached 0.8 accuracy using this code.
The provided ‘final’ Jupyter notebook uses a train:test split of 3461:470 (88:12)
Notebook excerpt outlining training and test data accuracy
Training data accuracy
Test data accuracy

## Slide 10
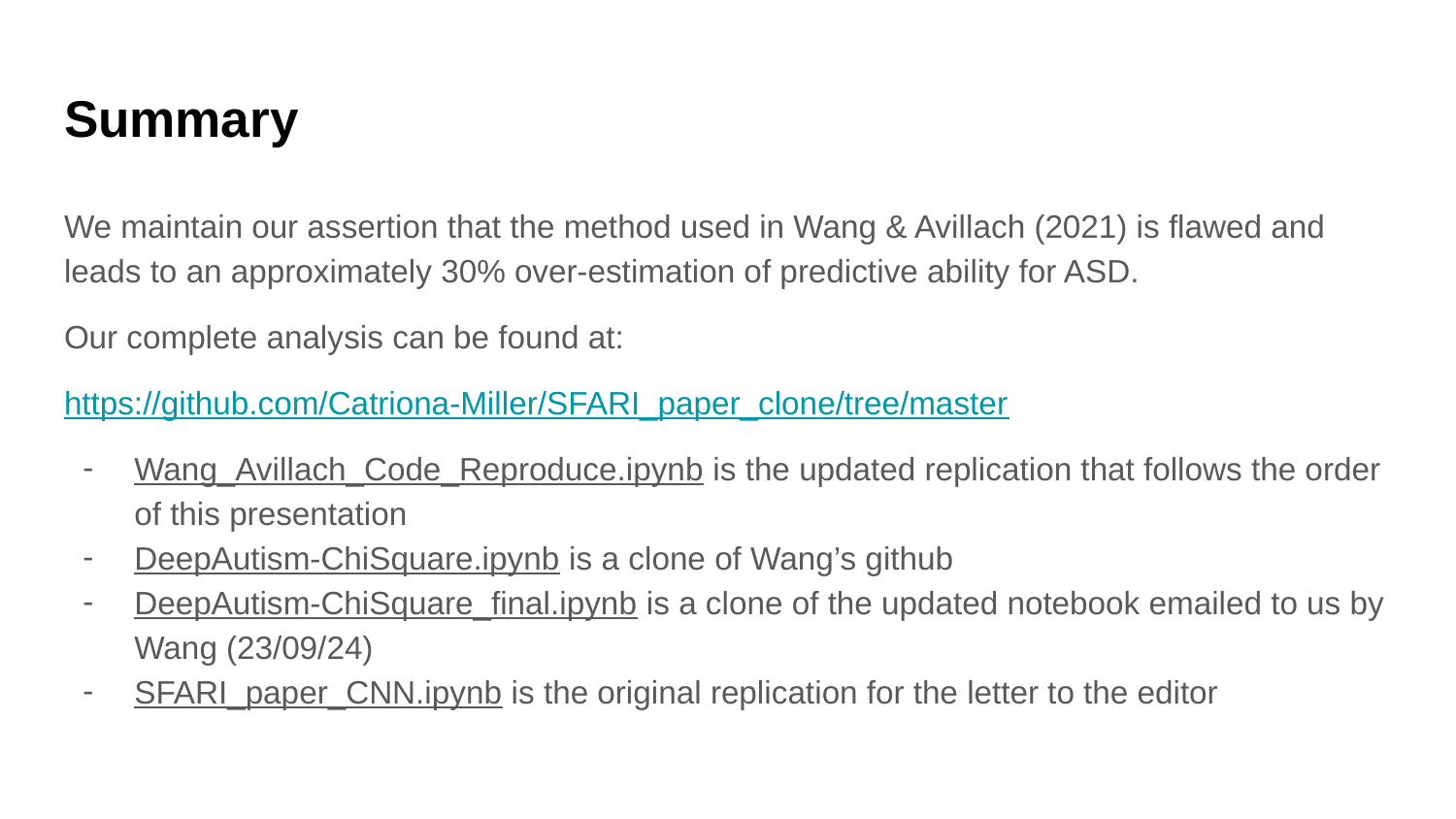

# Summary
We maintain our assertion that the method used in Wang & Avillach (2021) is flawed and leads to an approximately 30% over-estimation of predictive ability for ASD.
Our complete analysis can be found at:
https://github.com/Catriona-Miller/SFARI_paper_clone/tree/master
Wang_Avillach_Code_Reproduce.ipynb is the updated replication that follows the order of this presentation
DeepAutism-ChiSquare.ipynb is a clone of Wang’s github
DeepAutism-ChiSquare_final.ipynb is a clone of the updated notebook emailed to us by Wang (23/09/24)
SFARI_paper_CNN.ipynb is the original replication for the letter to the editor

## Slide 11
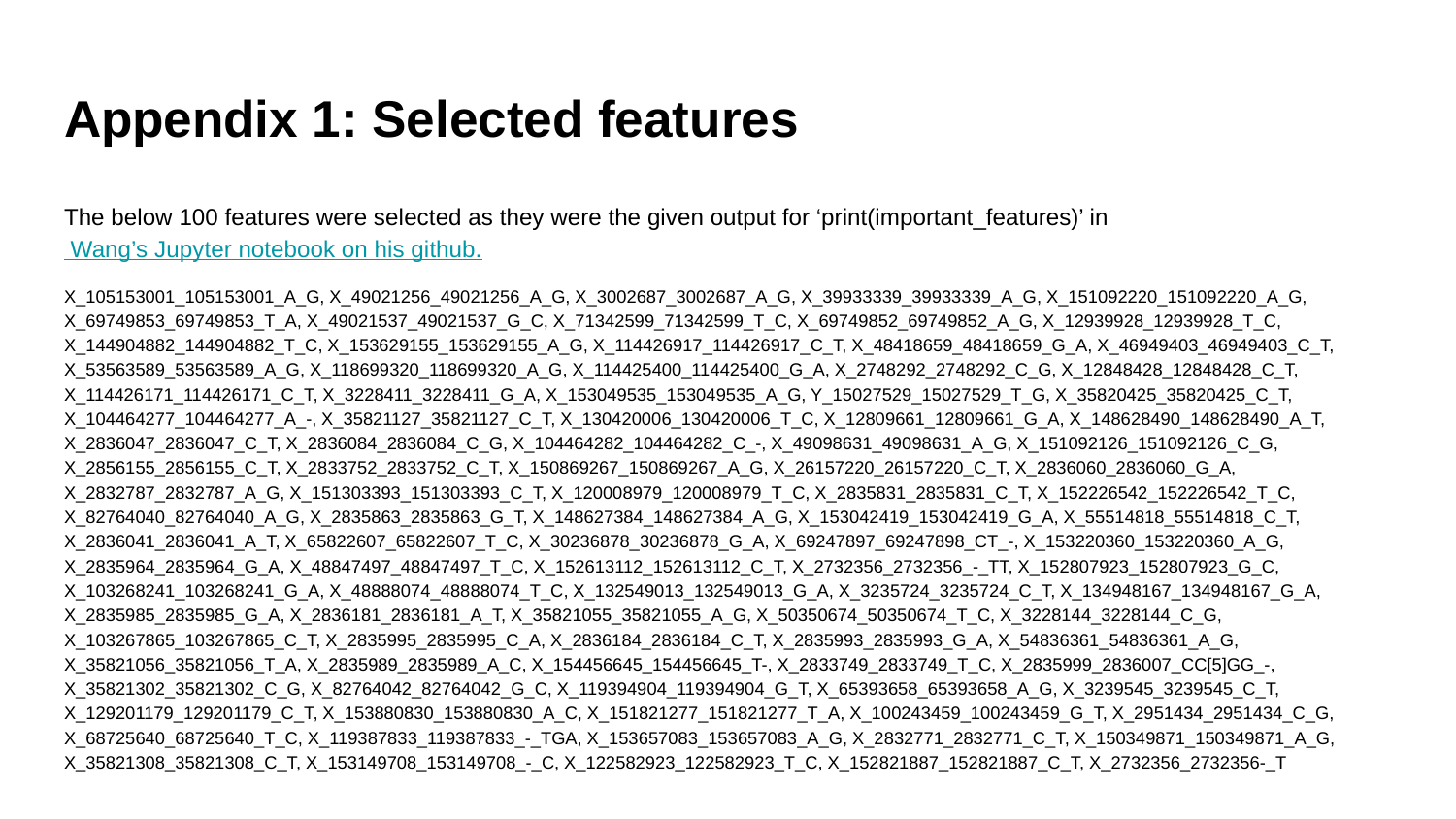

# Appendix 1: Selected features
The below 100 features were selected as they were the given output for ‘print(important_features)’ in Wang’s Jupyter notebook on his github.
X_105153001_105153001_A_G, X_49021256_49021256_A_G, X_3002687_3002687_A_G, X_39933339_39933339_A_G, X_151092220_151092220_A_G, X_69749853_69749853_T_A, X_49021537_49021537_G_C, X_71342599_71342599_T_C, X_69749852_69749852_A_G, X_12939928_12939928_T_C, X_144904882_144904882_T_C, X_153629155_153629155_A_G, X_114426917_114426917_C_T, X_48418659_48418659_G_A, X_46949403_46949403_C_T, X_53563589_53563589_A_G, X_118699320_118699320_A_G, X_114425400_114425400_G_A, X_2748292_2748292_C_G, X_12848428_12848428_C_T, X_114426171_114426171_C_T, X_3228411_3228411_G_A, X_153049535_153049535_A_G, Y_15027529_15027529_T_G, X_35820425_35820425_C_T, X_104464277_104464277_A_-, X_35821127_35821127_C_T, X_130420006_130420006_T_C, X_12809661_12809661_G_A, X_148628490_148628490_A_T, X_2836047_2836047_C_T, X_2836084_2836084_C_G, X_104464282_104464282_C_-, X_49098631_49098631_A_G, X_151092126_151092126_C_G, X_2856155_2856155_C_T, X_2833752_2833752_C_T, X_150869267_150869267_A_G, X_26157220_26157220_C_T, X_2836060_2836060_G_A, X_2832787_2832787_A_G, X_151303393_151303393_C_T, X_120008979_120008979_T_C, X_2835831_2835831_C_T, X_152226542_152226542_T_C, X_82764040_82764040_A_G, X_2835863_2835863_G_T, X_148627384_148627384_A_G, X_153042419_153042419_G_A, X_55514818_55514818_C_T, X_2836041_2836041_A_T, X_65822607_65822607_T_C, X_30236878_30236878_G_A, X_69247897_69247898_CT_-, X_153220360_153220360_A_G, X_2835964_2835964_G_A, X_48847497_48847497_T_C, X_152613112_152613112_C_T, X_2732356_2732356_-_TT, X_152807923_152807923_G_C, X_103268241_103268241_G_A, X_48888074_48888074_T_C, X_132549013_132549013_G_A, X_3235724_3235724_C_T, X_134948167_134948167_G_A, X_2835985_2835985_G_A, X_2836181_2836181_A_T, X_35821055_35821055_A_G, X_50350674_50350674_T_C, X_3228144_3228144_C_G, X_103267865_103267865_C_T, X_2835995_2835995_C_A, X_2836184_2836184_C_T, X_2835993_2835993_G_A, X_54836361_54836361_A_G, X_35821056_35821056_T_A, X_2835989_2835989_A_C, X_154456645_154456645_T-, X_2833749_2833749_T_C, X_2835999_2836007_CC[5]GG_-, X_35821302_35821302_C_G, X_82764042_82764042_G_C, X_119394904_119394904_G_T, X_65393658_65393658_A_G, X_3239545_3239545_C_T, X_129201179_129201179_C_T, X_153880830_153880830_A_C, X_151821277_151821277_T_A, X_100243459_100243459_G_T, X_2951434_2951434_C_G, X_68725640_68725640_T_C, X_119387833_119387833_-_TGA, X_153657083_153657083_A_G, X_2832771_2832771_C_T, X_150349871_150349871_A_G, X_35821308_35821308_C_T, X_153149708_153149708_-_C, X_122582923_122582923_T_C, X_152821887_152821887_C_T, X_2732356_2732356-_T

## Slide 12
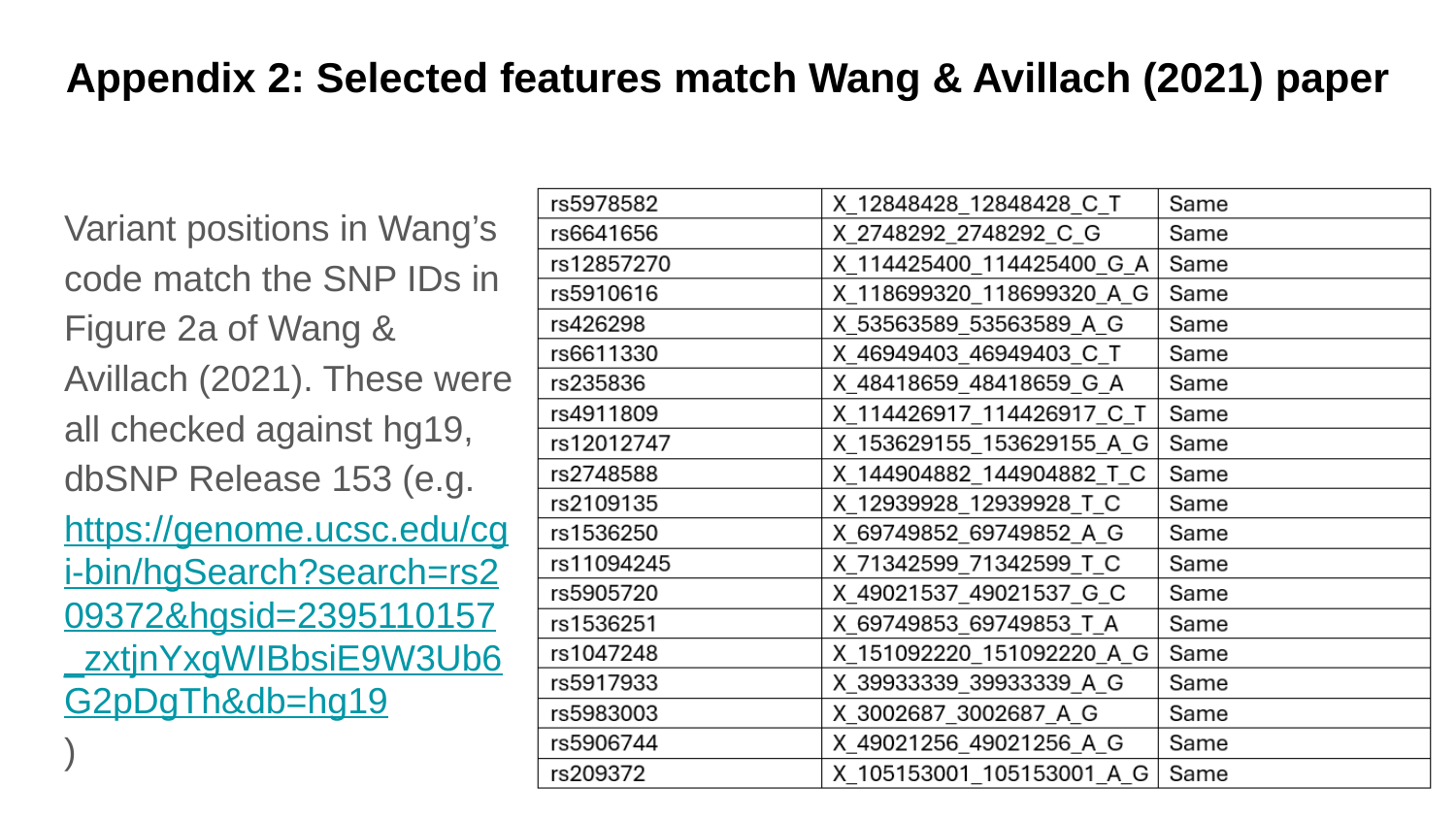

# Appendix 2: Selected features match Wang & Avillach (2021) paper
Variant positions in Wang’s code match the SNP IDs in Figure 2a of Wang & Avillach (2021). These were all checked against hg19, dbSNP Release 153 (e.g. https://genome.ucsc.edu/cgi-bin/hgSearch?search=rs209372&hgsid=2395110157_zxtjnYxgWIBbsiE9W3Ub6G2pDgTh&db=hg19)
